# Intussusception

**Published:** 1876-07

**Authors:** 


### INTUSSUSCEPTION.

Turn a stocking wrongside out part way, then turn it up with the toe downwards, any weight or force downwards only pushes the toe still further down. That is what we mean, by that speedily and terribly fatal disease, "Intussusception of the Bowels." It is an upper portion of the bowels pushing down with a kind of pocket within a lower portion. In such case nothing passes through the body, and death is inevitable. One of the most eminent and admired men of South Carolina perished with that disease; and so, most probably, within a short time, the Rev. Daniel H. of the New York Annual Conference of the Wesleyan Methodist Church, a worthy clergyman of great industry and decision of character.

It is not the design of this journal ever to advise medicinal means, that is the province of the regular physician. But when ailments can be removed by any article of food, or by air, exercise, and the like, we feel free to recommend them. In a case of "*intussusception*," where quicksilver, croton oil, and other powerful means had failed, and for nine days the patient had nothing to pass through the bowels, the drinking of a pint of hot molasses, without stopping, gave immediate and permanent relief, without vomiting, nausea, or anything of the kind. *How* it acted, is worthy of investigation. The facts we know to have been as stated, and we hope our exchanges will give this a wide circulation. An attack of "intussusception" comes on so suddenly, we know so little of its causes, it is attended generally with such intense suffering, and relief is so seldom obtained, that physicians generally regard it with hopeless alarm.

Looking over the whole subject of servants, we should consider their ignorance, and pity and bear with them; treat them courteously; keep them at a respectful distance; require system and punctuality in all things. Let them feel that your will is law, that no injunction is to be given beyond the second time, and that their whole duty to you will be exacted by you, as much as the last cent of their wages is exacted by them. Patiently instruct them, and always speak kindly, or if in reproof, even that may be mildly done; and when they leave, let certificates be given, whose literal truth shall do even-handed justice to the one who has left you, as well as to the household where she may next find employment. Such a course, would work a domestic revolution within a year.

